# A Multi-Network Comparative Analysis of Transcriptome and Translatome Identifies Novel Hub Genes in Cardiac Remodeling

**DOI:** 10.3389/fgene.2020.583124

**Published:** 2020-11-16

**Authors:** Etienne Boileau, Shirin Doroudgar, Eva Riechert, Lonny Jürgensen, Thanh Cao Ho, Hugo A. Katus, Mirko Völkers, Christoph Dieterich

**Affiliations:** ^1^Section of Bioinformatics and Systems Cardiology, Klaus Tschira Institute for Integrative Computational Cardiology, Heidelberg, Germany; ^2^Department of Internal Medicine III (Cardiology, Angiology, and Pneumology), University Hospital Heidelberg, Heidelberg, Germany; ^3^DZHK (German Centre for Cardiovascular Research), Partner Site Heidelberg/Mannheim, Berlin, Germany

**Keywords:** cardiovascular, cardiac hypertrophy, transcription/RNA-seq, translation/Ribo-seq, co-expression networks

## Abstract

Our understanding of the transition from physiological to pathological cardiac hypertrophy remains elusive and largely based on reductionist hypotheses. Here, we profiled the translatomes of 15 mouse hearts to provide a molecular blueprint of altered gene networks in early cardiac remodeling. Using co-expression analysis, we showed how sub-networks are orchestrated into functional modules associated with pathological phenotypes. We discovered unappreciated hub genes, many undocumented for their role in cardiac hypertrophy, and genes in the transcriptional network that were rewired in the translational network, and associated with semantically different subsets of enriched functional terms, such as *Fam210a*, a novel musculoskeletal modulator, or *Psmd12*, implicated in protein quality control. Using their correlation structure, we found that transcriptome networks are only partially reproducible at the translatome level, providing further evidence of post-transcriptional control at the level of translation. Our results provide novel insights into the complexity of the organization of *in vivo* cardiac regulatory networks.

## 1. Introduction

Exercise- and disease-induced cardiac growth are associated with different molecular profiles and differ in the signaling pathways that drive remodeling; yet both are characterized by an increase in size of cardiomyocytes, sarcomerogenesis, and overall increase in heart-weight-to-body-weight (HW/BW) ratio (Shimizu and Minamino, 2016). While adaptive exercise-induced hypertrophy allows the heart to maintain an adequate cardiac output with improved contractility, pathological hypertrophy is a maladaptive response, concurring with irreversible changes (e.g., cardiomyocyte loss, fibrosis, reduced cardiac function), and typically progressing to heart failure (Shimizu and Minamino, 2016; Bernardo et al., 2018).

Little is known about the molecular mechanisms controlling physiological hypertrophy, particularly from a multi-omics systems biology perspective. Yet our understanding of the *in vivo* transition from adaptive hypertrophy to cardiac dysfunction has important clinical implications (Boström et al., 2010). Only recently have post-transcriptional regulatory networks been uncovered that are of central importance for morphological remodeling in fibrosis (Chothani et al., 2019), or for modulating the early reponse to cardiac stress (Doroudgar et al., 2019).

In this study, we adopt a systems biology approach to integrate multi-omics data through the use of co-expression networks to highlight higher-order relationships among gene programs that are expressed in the heart *in vivo* under growth stimuli. In such networks, genes are connected if there is a significant co-expression relationship between them (Langfelder and Horvath, 2008). Modules or sub-networks represent clusters of genes with related function or involved in common processes or pathways. Our analysis of 15 mouse left ventricular tissues from experimental models of exercise- and disease-induced cardiac hypertrophy showed, for the first time, the organization of the transcriptome and translatome into networks of biologically meaningful clusters of co-expressed genes. By correlating module expression and disease phenotypes, we were able to show the synchronized expression dynamics of genes encoding extracellular matrix, and cytoskeletal proteins, and a diminished contribution of electron transport complex genes, genes associated with oxidative phosphorylation and mitochondrial function. While concerted dynamic changes were observed in both transcriptome and translatome networks, transcriptome networks were only partially reproducible at the translatome level, reflecting the existence of RNA- or Ribo-specific modules. In contrast to differential expression analysis, co-expression and rewiring analysis led us to the identification of yet uncharacterized candidate genes, key to organizing the behavior of transcriptome and translatome networks. In particular, our results uncovered *Fam210a*, a novel musculoskeletal modulator, which we hypothesize to regulate the expression of mitochondrial encoded genes; and *Psmd12*, a regulatory subunit of the 26S proteasome, whose deregulation may act as a pathogenic factor compromising protein quality control in cardiomyocytes.

## 2. Materials and Methods

### 2.1. Experimental Models

We acquired data from experimental models of pathological cardiac hypertrophy (transverse aortic constriction or TAC) and swimming-induced physiological hypertrophy. TAC (27 gauge needle) surgery was performed as previously described (Völkers et al., 2013), and animals were sacrificed after 2 days (*n* = 4) and 2 weeks (*n* = 5). For exercise training in the physiological hypertrophy model, mice swam regularly in a water tank for either 2 days (*n* = 3) or 2 weeks (*n* = 3) (Evangelista et al., 2003). The experiments were performed in 9-week-old male C57Bl6/N mice using the RiboTag system (Doroudgar et al., 2019; Kmietczyk et al., 2019). All animal experimental procedures were reviewed and approved by the Institutional Animal Care and Use Committees at the Animal Experiment Review Board of the government of the state of Baden-Württemberg, Germany.

### 2.2. Preparation of Sequencing Libraries

Mice were sacrificed, and their hearts were excised, washed in PBS containing 100 μg/ml cycloheximide (CHX), and snap frozen in liquid nitrogen. Left ventricular tissue was homogenized using a tissue homogenizer in 5 volumes of ice-cold polysome buffer (20 mM Tris pH 7.4, 10 mM MgCl, 200 mM KCl, 2 mM DTT, 1% Triton X-100, 1U DNase/μl) containing 100 μg/ml CHX. Ribo-seq and RNA-seq libraries were prepared for each biological replicate from the identical lysate. Ribosome protected fragments (RPFs) were generated after immunoprecipitation of cardiac myocyte-specific polysomes with anti-HA magnetic beads after treating the lysate with RNase I (Ambion). Libraries were generated according to the mammalian Ribo-seq kit (Illumina), and sequenced on the HiSeq 2000 platform using a 50-bp sequencing chemistry.

### 2.3. The RiboTag System

In the RiboTag mouse, the exon 4 of the Rpl22 gene is flanked by Loxp recombination sites, followed by an HA-tagged exon 4. When the RiboTag mouse is crossed to a Cre driver mouse, the Cre recombinase enzyme is activated resulting in the removal of the LoxP-flanked wild type Rpl22 exon 4 and replacement with the HA-tagged Rpl22 exon 4, which is incorporated into the ribosome particle. In mouse hearts, a cell-specific promotor (Myosin heavy chain, α isoform, or αMHC, encoded by the Myh6 gene) drives the expression of Cre which induces cardiomyocyte-specific HA-tagged ribosomes. RiboTag mice were purchased from Jackson Laboratory (JAX ID 011029) and bred to the αMHC-Cre mice line to obtain homozygous mice expressing Rpl22-HA in cardiomyocytes.

### 2.4. Detecting Active Translation

Translation prediction using Ribo-seq data was performed with Rp-Bp v2.0 (Malone et al., 2017), based on Ensembl release 96. We used evidence from uniquely mapped reads and periodic fragment lengths only. For each sample, the fragment lengths and ribosome P-site offsets were determined from a metagene analysis using the automatic Bayesian selection of read lengths and ribosome P-site offsets (BPPS). The final list of translation events includes, in addition to annotated open reading frames (ORFs), ORFs with evidence of translation outside of annotated coding sequences (Supplementary Table 1). For the analyses, translation in non-coding regions as well as variants of canonical coding sequences were discarded (Supplementary Figures 1, 2). We required ORFs to have a minimum length of 3 aa and more than 10 in-frame P-sites. The final list of translation events was further filtered to only include ORFs that were predicted in at least three samples and whose host gene was also annotated in APPRIS (Rodriguez et al., 2018), resulting in 9,129 unique genes (Supplementary Table 2).

### 2.5. Sequencing Data Alignment

Adapters removal and quality filtering was done with flexbar v3.0.3 (Dodt et al., 2012) using standard filtering parameters implemented in Rp-Bp. Reads aligning to a custom bowtie2 v2.3.0 (Langmead and Salzberg, 2012) ribosomal index were discarded. Remaining reads were then aligned in genomic coordinates to the mouse genome (GRCm38.p6) with STAR v2.5.3a (Dobin et al., 2013). For the RNA-seq data, reads were trimmed from the 3' end after adapter removal, to match the maximum periodic fragment length, as determined with the BPPS method, for each sample. Finally, abundance estimates and read count to coding sequences were obtained using HTSeq-count (Anders et al., 2014), taking into account the strand-specific protocols.

### 2.6. Constructing Gene Co-expression Networks

Read counts to coding sequences were used, only including genes that were considered to be translated (9,129 genes), as explained in the section 2.4. We removed low variance genes and genes with the lowest sequencing-depth normalized average expression (first centile). From these, 7,976 genes with the highest connectivity were clustered on the basis of topological overlap (TO) to identify patterns of co-expression, using the WGCNA R package (Langfelder and Horvath, 2008, 2012) (Supplementary Table 3). The network construction was done separately for Ribo-seq and RNA-seq data, on this common set of genes. We first applied a regularized log transformation, and corrected for batch effects, where applicable (Johnson et al., 2006). Weighted adjacencies were defined based on signed co-expression similarity using biweight midcorrelation and a soft thresholding power of β = 18 (for both RNA-seq and Ribo-seq). For each network, a reference TO matrix was first calculated. To produce robust and reproducible clusters, we then performed bootstrap-resampling (*n* = 100) and computed the TO matrix for each of the resampled networks. In each case, resampling was done within the physiological (swim) or the pathological (TAC) group. The final consensus TO matrix was defined as the median of all scaled TO matrices, and used as input for hierarchical clustering. The consensus TO matrix can be viewed as a “smoothed” version of the adjacency matrix. Network modules whose eigengenes were highly correlated were merged, and characterized by their eigengene expression and significance. To validate module membership, we applied *post-hoc* resampling (*n* = 100) by subsetting TO of random modules matched by size with respect to the consensus TO for every module. A one-proportion Z-test was used to assess whether the mean TO of the random modules was higher than that of the module assigned by the hierarchical clustering and merging algorithm.

The module membership is defined as the correlation (biweight midcorrelation) between eigengene and gene expression values, and measures the importance of a gene within a cluster. Gene significance is defined as the correlation (biweight midcorrelation) between genes and biological traits or disease association. To create indicators for level contrasts, e.g., pathological vs. physiological, TAC 2d vs. swim 2d, or TAC 2w vs. swim 2w, categorical variables were binarized as input for correlation. The pathological model includes all TAC samples, and the physiological model includes all swim samples. For binary/discrete variable correlation, biweight midcorrelation was replaced by the standard Pearson correlation. Hub genes were defined as genes with the highest intramodular connectivity. We ranked genes in each module and selected as hub genes those having a number of interactions greater than two standard deviations above the average connectivity found in a given module, i.e., with a Z-score > 2.

Preservation statistics were derived using the RNA-seq network as a reference, using the correlation structure of the networks. We used the *medianRank* statistic, defined as the mean of the observed density and connectivity statistics, and the *Z*_*summary*_, defined as the mean of *Z*-scores computed for density and connectivity measures (Langfelder et al., 2011). The *medianRank* was used to compare relative preservation among clusters. The *Z*_*summary*_ was used to assess the significance of observed statistics by distinguishing preserved from non-preserved clusters via permutation testing (*n* = 100).

Differentially connectivity (DC) was defined as the $Log_{10}\left(k_{in}^{RNA}/k_{in}^{Ribo}\right)$. It is not a robust measure, and it is based uniquely on intramodular connectivity. A gene was DC if this ratio was greater than two standard deviations above the average across all genes. All results are found in Supplementary Table 4.

### 2.7. Dynamic Neighborhoods Score

The Dn score, or dynamic neighborhoods score, of a given gene is calculated based on the variance of the state-space adjacency matrix over the network states (RNA-seq and Ribo-seq), relative to the mean centroid, as $\text{Dn}=\sum_{i=1}^{RNA,Ribo}d{\left(V_{i},centroid\right)}^{2}$, where *d* is the Euclidean distance, and **V_i_** is a vector of genes in the RNA-seq and Ribo-seq networks (Goenawan et al., 2016). To calculate the Dn score, we used the consensus TO matrix, thresholded at 0.1 (all interactions below this threshold were considered inexistant).

### 2.8. Differential Translational-Efficiency Analysis

Differential translational-efficiency analysis was performed using DESeq2 (Love et al., 2014). The calculation of change in translational efficiency was done using an interaction term (~assay + condition + assay:condition) with a likelihood-ratio test, accounting for variance and level of expression. Regulation status of a gene at the transcriptional and/or translational level can be integrated using the fold changes from standard Wald test (RNA-seq, Ribo-seq) and the likelihood-ratio test (translational efficiency). All analyses were performed on the same set of genes used as input for network clustering (*n* = 7,976). We used genome-wide significance threshold of FDR < 0.05, and a fold change (FC) of log_2_(1.2). All results are found in Supplementary Table 7.

### 2.9. Gene Ontology (GO) Enrichment

GO enrichment (The Gene Ontology Consortium, 2018) to assign functional annotation to modules were performed with topGO v2.34.0 (Alexa and Rahnenfuhrer, 2018). To define the relevant gene sets corresponding to each clusters, we considered hub genes and genes with strong module membership and significance for a given trait (heart-weight-to-body-weight ratio, pathological vs. physiological, TAC2d vs. swim2d, or TAC2w vs. swim2w). The latter were identified by taking the upper quartile of genes with the highest module membership and gene significance for a trait having the highest correlation between absolute values of module membership and gene significance. The universe of genes consisted of all translated genes (*n* = 9,129). All results are found in Supplementary Table 5.

## 3. Results

### 3.1. Exercise- vs. Disease-Induced Cardiac Remodeling

To characterize stress-induced cardiac remodeling and the response to acute and chronic pressure overload, we used the swim model (Wang et al., 2010) and the transverse aortic constriction (TAC) model (Rockman et al., 1991). The swim model is referred to as the physiological or the healthy model, and represents exercise-induced hypertrophy. The TAC model is referred to as the pathological model, and represents disease-induced hypertrophy.

**Figure 1:**
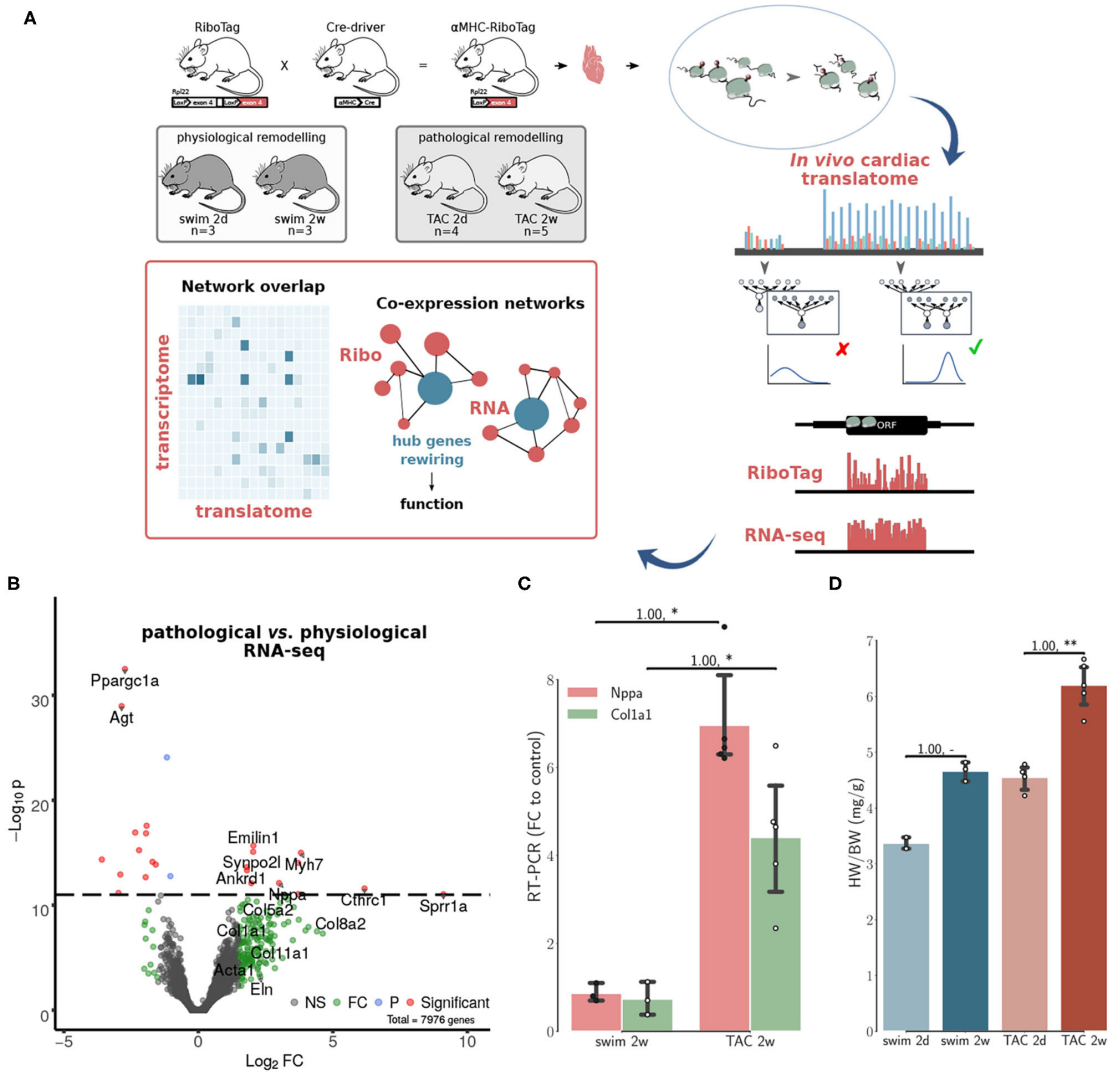
Co-expression networks of transcriptional and translational regulation in cardiac remodeling. **(A)** The RiboTag system is used to find the cardiac translatome of 15 animals from two models at two time points. A final set of translated genes is inferred using graphical Bayesian models. Co-expression networks are then constructed separately for *in vivo* Ribo-seq (translatome from RiboTag data) and RNA-seq (transcriptome) data. The networks were used to identify hub genes associated with cardiac remodeling, and how changes in connectivity (network rewiring) are associated with differential functionality in transcriptome vs. translatome, see also Supplementary Figure 3. **(B)** Volcano plot displaying the distribution of all genes with relative abundance (log_2_ RNA-seq pathological vs. physiological) plotted against significance level, showing significantly increased and decreased genes during *in vivo* pathological stress. Top genes (red) are highlighted. A number of genes (green) significant at a threshold of 0.05 are also highlighted. NS, Non-significant; FC, Fold change only significant; P, *p*-value only significant; Significant, FC+P. **(C)** RT-PCR fold changes relative to control animals (2w) for two important markers of hypertrophy and fibrosis, corroborating evidence from RNA-seq shown in **(B)**. **(D)** Heart-weight-to-body-weight ratio (HW/BW) after 2d and 2w of exercise in the physiological model, 2d and 2w after transverse aortic constriction (TAC) surgery. For **(C,D)**, N≥3 at each time point. Significance was measured using Mann–Whitney *U*-test, at a threshold of * < 0.05 (** < 0.01, *p*-values may be affected by the small sample size, for this reason we also report effect size using non-directional rank-biserial correlation). For **(D)** only, Kruskall–Wallis test *H* = 10.9, *p* = 0.01.

To study co-expression networks during cardiac remodeling, we used *in vivo* Ribo-seq and RNA-seq libraries from 15 mouse hearts (Figure 1A). We used the RiboTag approach to capture the cardiac translatome. Cardiomyocyte-specific analysis of ribosome protected fragments was achieved after affinity purification using the RiboTag mouse (Doroudgar et al., 2019). To catalog translation events, we performed an unsupervised search for actively translated open reading frames (ORFs) using Rp-Bp (Malone et al., 2017) (Supplementary Figures 1, 2). The final list of translated genes was used as background for co-expression and differential expression analyses. Co-expression networks were constructed separately for RNA-seq (transcriptome) and Ribo-seq (translatome) count data and used to identify hub genes associated with cardiac remodeling (Supplementary Figure 3). All translated ORFs used in this study, RNA-seq and Ribo-seq read counts can be found as Supporting Information and Supplementary Tables 2, 3.

We monitored the acute response at an intermediate time point (2 days after TAC), and a chronic time point (2 weeks after TAC), when cellular and molecular remodeling has occurred, but cardiac function is preserved (Doroudgar et al., 2019). Matching time points were monitored in the physiological model (swim at 2 days and 2 weeks). At the RNA-seq level only, we observed an upregulation of *Nppa*, the fetal isoform of myosin heavy chain (*Myh7*) (Taegtmeyer et al., 2010), clinically relevant genes such as *Ankrd1* or *Synpo2l* (Ling et al., 2017; van Eldik et al., 2017), as well as a number of genes implicated in tissue remodeling (Figure 1B). These observations were corroborated with RT-PCR results for two markers of hypertrophy and fibrosis (Figure 1C). Increased HW/BW ratios were detected after 2 weeks in the swim and TAC models, with a larger increase in the pathological model (Figure 1D). Taken together, these results are consistent with graded, pathological cardiac hypertrophy in the TAC model. In the physiological model, we did not observe fetal gene re-expression, typically associated with metabolic remodeling in a variety of pathophysiologic conditions (Taegtmeyer et al., 2010). There was a significant increase in *Ppargc1a*, a master regulator of mitochondrial biogenesis associated with physiological hypertrophy (Boström et al., 2010), and *Agt*, key component of the renin-angiotensin system (RAS), suggesting that the swim model did not induce a pathological hypertrophy phenotype, but instead improved the cellular energetics of the heart.

### 3.2. Co-expression Networks of Cardiac Remodeling

**Figure 2:**
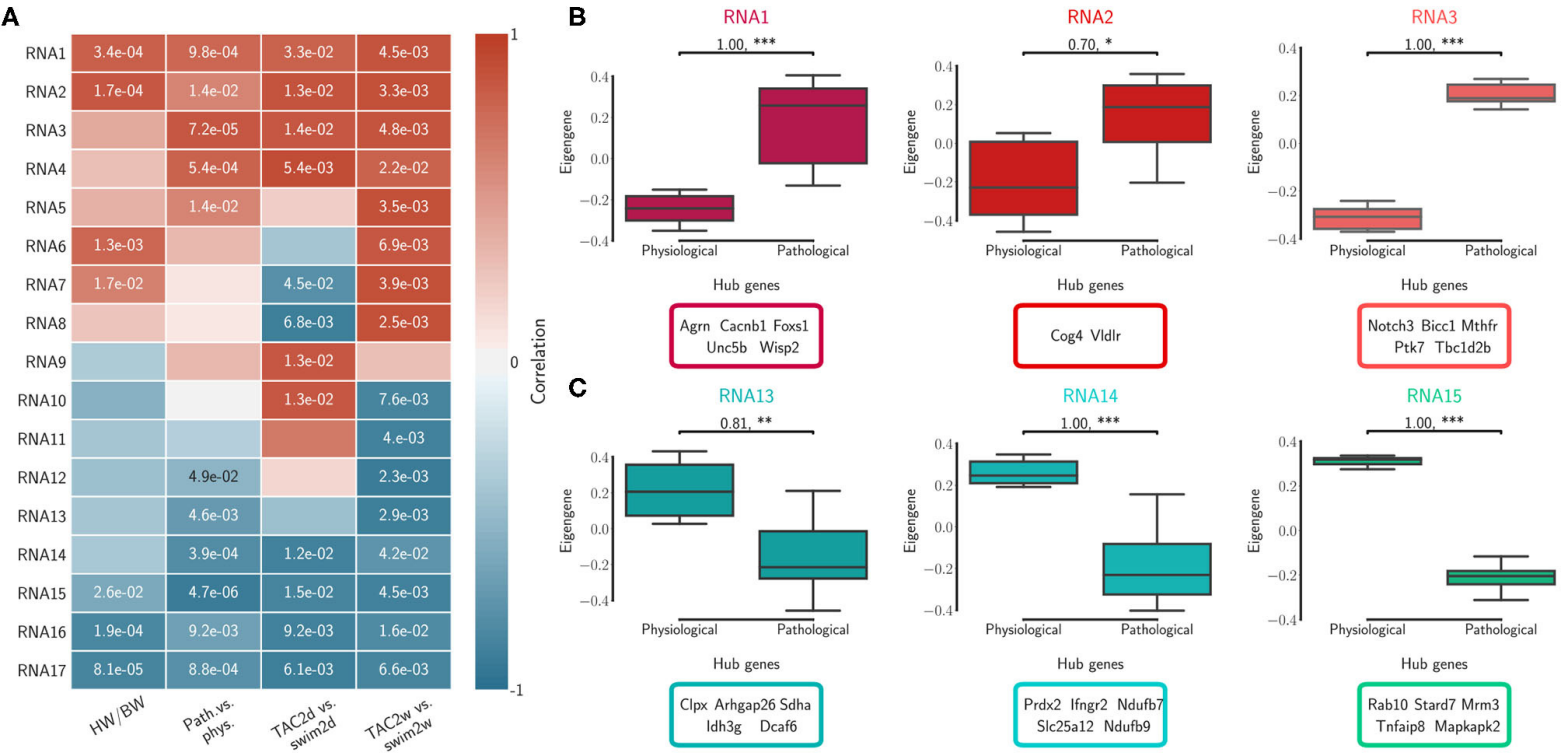
Transcriptome (RNA) network modules were clustered to assess relatedness based on correlation of co-expressed eigengenes. **(A)** Biweight midcorrelation and Student correlation *p*-values between eigengene module expression and disease association as well as heart-weight-to-body-weight ratio. **(B)** The first three module expression profiles and top ranked hub genes that are positively correlated with pathological cardiac remodeling. **(C)** The first three module expression profiles and top ranked hub genes that are negatively correlated with pathological cardiac remodeling (i.e., positively correlated with the physiological model). Significance was measured using a one-sided Mann-Whitney *U*-test, at a threshold of * < 0.05 (** < 0.01, *** < 0.001, *p*-values may be affected by the small sample size, for this reason we also report effect size using non-directional rank-biserial correlation). Up to five hub genes are shown.

**Figure 3:**
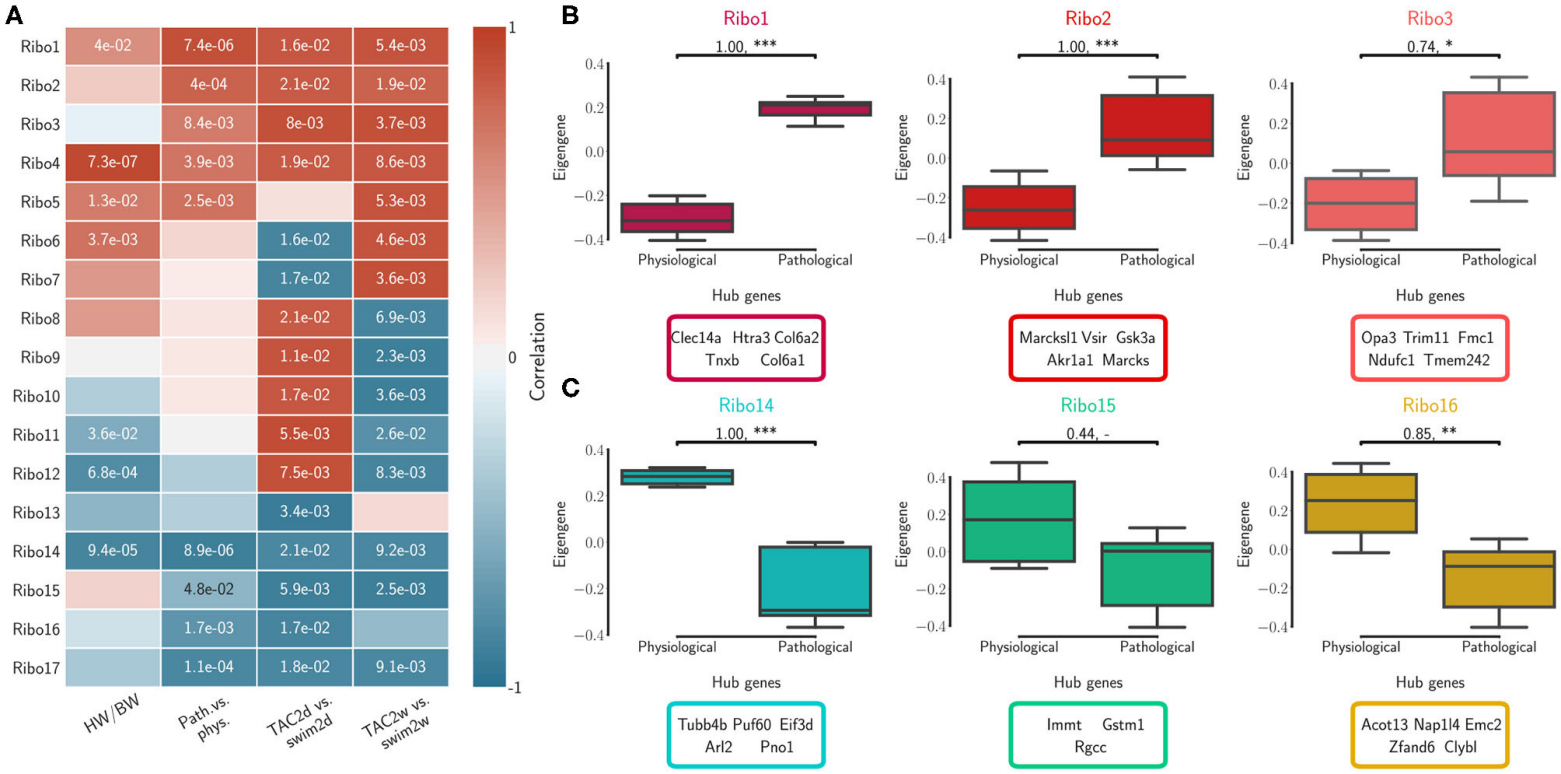
Translatome (Ribo) network modules were clustered to assess relatedness based on correlation of co-expressed eigengenes. **(A)** Biweight midcorrelation and Student correlation *p*-values between eigengene module expression and disease association as well as heart-weight-to-body-weight ratio. **(B)** The first three module expression profiles and top ranked hub genes that are positively correlated with pathological cardiac remodeling. **(C)** The first three module expression profiles and top ranked hub genes that are negatively correlated with pathological cardiac remodeling (i.e., positively correlated with the physiological model). Significance was measured using a one-sided Mann-Whitney *U*-test, at a threshold of * < 0.05 (** < 0.01, *** < 0.001, *p*-values may be affected by the small sample size, for this reason we also report effect size using non-directional rank-biserial correlation). Up to five hub genes are shown.

We calculated topological overlap and clustered genes, identifying independently 17 distinct co-expression modules for each of the RNA-seq and Ribo-seq networks (Supplementary Table 4). The modules were labeled in order from RNA1 to RNA17, and from Ribo1 to Ribo17, using unsupervised hierarchical clustering based on co-expression correlation with disease association (Figures 2A, 3A). Our analysis revealed how gene expression programs in the heart are organized differently in transcriptome and translatome space into modules, or sub-networks, of highly connected genes. Gene Ontology (GO) enrichment analysis suggest that significant genes in a number of modules localize to common cellular components, such as the extracellular matrix (ECM) and associated proteins (RNA1, RNA7, Ribo1, Ribo2, and Ribo4), the cytoskeleton, related membrane ruffling (RNA4 and RNA6) and the cell cortex (Ribo8, Ribo10), the Golgi apparatus (RNA2), the nucleosome (Ribo12), or the various sub-compartments of the mitochondrion (RNA13, RNA14, RNA15, Ribo13, and Ribo16) (Supplementary Table 5). These modules had one or more related molecular function or were associated with shared biological processes.

#### 3.2.1. Correlation With the Cardiac Pathophysiology of Remodeling

We calculated the module correlations to disease association and HW/BW ratios, and clustered them to identify sub-networks associated with patho-physiological features and pre-clinical symptoms of cardiac hypertrophy. Five RNA modules and five Ribo modules had a positive correlation to the pathological model and to increased HW/BW ratio, and almost all were significant (RNA1 to RNA5, Ribo1 to Ribo5, Figures 2A, 3A). We also observed a negative correlation to the pathological model (i.e., a positive correlation to the physiological model) in five RNA modules and, to a moderate extent, in four Ribo modules (RNA13 to RNA17, Ribo14 to Ribo17). RNA1 to RNA5 (Ribo1 to Ribo5) were activated after pressure overload in the pathological model, whereas RNA13 to RNA17 (Ribo14 to Ribo17) were repressed, when looking at the module eigengenes (Figures 2B,C, 3B,C, and Supplementary Figures 4A,B 5A,B). An eigengene is a representative of the standardized module expression values across all samples. Eigengenes have been largely regarded as robust biomarkers (Oldham et al., 2008; Johnson et al., 2018; Zhang et al., 2018; Di et al., 2019). The strong association between RNA1 to RNA5 (Ribo1 to Ribo5), on the one hand, and that of RNA13 to RNA17 (Ribo14 to Ribo17), on the other hand, suggests a synchronized expression dynamics characterized by an increased role of genes encoding ECM and cytoskeletal proteins, and a diminished or altered contribution from mitochondrial translation, metabolic pathways of carbohydrate, fat, and protein metabolism, as well as oxidative phosphorylation. RNA6 to RNA12 (Ribo6 to Ribo13) showed a dynamic association pattern to either the 2d or the 2w time points, uncovering the transcriptional and translational heterogeneity in response to pressure overload (Figures 2A, 3A, and Supplementary Figures 4C, 5C). The significance of these associations was highly consistent, and when we looked at the correlation between module membership and gene significance, we found that the strongest and most significant associations were for the top five modules, particularly for RNA (RNA1 to RNA5) (Supplementary Table 6). In addition, for modules RNA6 to RNA12, gene significance at 2w was more often and more strongly correlated with module membership, suggesting that driver genes are associated with the later time points. On the contrary, for most modules Ribo6 to Ribo13, the association was observed with the earlier time points, supporting a higher relative contribution of translational control at 2d, consistent with a rapid translational response to stress (Doroudgar et al., 2019). In each module, we also identified intramodular hub genes, which are highly co-expressed genes with respect to all other genes in the same module, and may thus function as key components of the hypertrophic response (Figures 2B,C, 3B,C, and Supplementary Figures 4, 5). Finally, markers of the fetal gene program were found in sub-networks correlated with the pathological models (Taegtmeyer et al., 2010) (Supplementary Table 4): *Nppa* (RNA1, Ribo1), *Nppb* (RNA3, Ribo7), or *Myh7* (RNA3, Ribo1). While *Myh6* was found in RNA15 and Ribo16, consistent with the observed known “gene switches,” we observed the presence of several other genes clustered in RNA1, Ribo4 or Ribo2 (*Myh7b, Myh10, Myh11*, and *Myh14*), and whose clinical significance has not yet been described in the context of cardiac hypertrophy. These switches were also observed for *Glut1* and *Glut4*: *Slc2a8* (RNA1, Ribo1), *Slc2a1* (RNA10, Ribo10), *Slc2a4* (RNA14, Ribo3), or *Slc2a12* (RNA15, Ribo17); and for *Myc*: *Mycn* (RNA1, Ribo4), and *Myc* (RNA17, Ribo10).

#### 3.2.2. Co-expression Networks Uncover Hub Genes Not Found by Differential Expression

Unsupervised hierarchical clustering based on hub gene expression showed that the top interacting genes serve as a molecular signature to differentiate physiological and pathological models of cardiac hypertrophy (Supplementary Figures 6A, 7A). We then compared these observations with results from differential translational-efficiency (DTE) and differential connectivity (DC) analyses (Supplementary Figure 8 and Supplementary Table 7 for extended DTE results). Although a large number of non-significant genes in DTE showed a higher DC between RNA-seq and Ribo-seq networks, hub genes remain relatively unchanged in DC (Supplementary Table 4). Many hub genes from modules with positive (RNA1 to RNA5, and Ribo1 to Ribo5) or negative correlation (RNA13 to RNA17, and Ribo14 to Ribo17) were up-/down-regulated in the pathological model, respectively (Supplementary Figure 6B). Similar observations were made considering the different time points and the varying correlations, using hub genes from modules RNA6 to RNA12, and Ribo6 to Ribo13 (Supplementary Figure 7B). While the number of differentially regulated genes is much larger, we found hub genes from co-expression correlation only that were not identified in DTE.

#### 3.2.3. Co-expression Networks Describe the Organization of the Heart Transcriptome and Translatome

**Figure 4:**
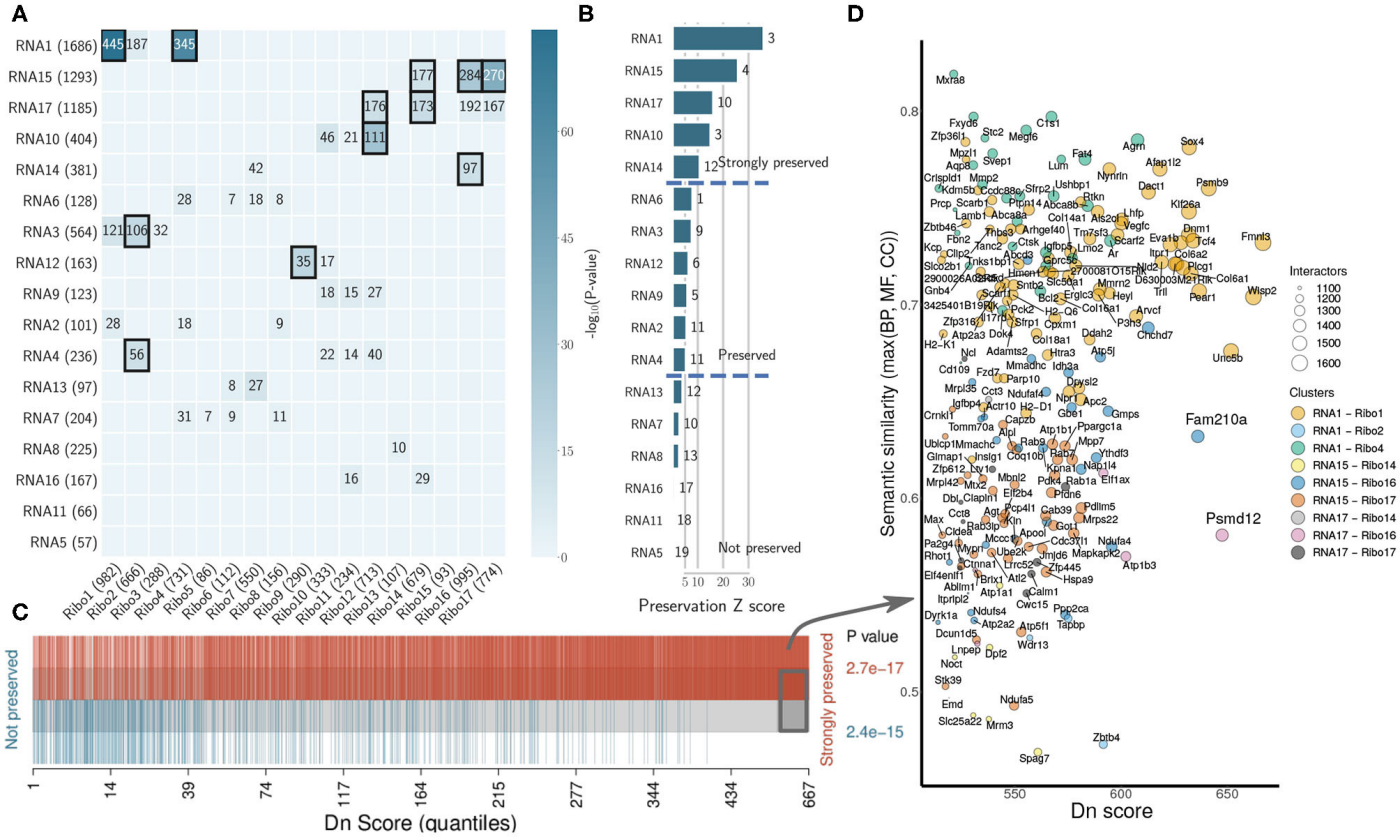
The organization of the heart transcriptome and translatome under cardiac remodeling. **(A)** Cross-tabulation of RNA-seq (rows) and Ribo-seq (columns) modules. Each row and column is labeled by the corresponding module and its size (number of genes). Counts of genes in the intersection is shown for modules sharing a significant overlap (*p* < 0.01, two-tailed Fisher's exact test). Overlap with *p* < 1e-10 are highlighted. **(B)** Preservation Z scores (mean of *Z*-scores computed for density and connectivity measures) for the RNA-seq modules in the Ribo-seq network. The vertical lines indicate the threshold for moderate preservation (5) and strong preservation (10). The *medianRank* statistic is indicated to the right of each bar. **(C)** Barcode plot showing the enrichment of genes based on their dynamic neighborhoods score (Dn score), compared to their association with modules that are either strongly or not preserved. Genes are ranked according to their Dn score, and colored based on their association. The gray box toward the right indicates genes for which Dn score is greater than two standard deviations above the average. To test whether genes belonging to preserved or not preserved clusters are highly ranked in terms of Dn score, the camera test from the limma R package was performed and *p*-values are shown. **(D)** Scatter plot of the most rewired genes (gray box in **C**). A semantic similarity analysis was performed for each ontology (BP, MF, and CC) and every gene using its immediate interactors in the RNA-seq and Ribo-seq networks, respectively, and the maximum semantic similarity is reported on the y-axis. The set of all translated genes was used as background. Dots are colored according to their cluster association, and the size represents the total number of interactors, or connected genes, in a hypothetical joint network combining the respective RNA-seq and Ribo-seq modules. Semantic similarity was calculated using the GOSemSim R package. Only genes in modules with significant overlap are reported. BP, Biological Process; MF, Molecular Function; CC, Cellular Component.

We investigated the degree of preservation between RNA network structure and Ribo co-expression network, and the amount of overlap between sub-networks. Preservation is based on density and connectivity measures (Langfelder et al., 2011), and uses the correlation structure of the networks to identify differences between RNA-seq and Ribo-seq. We identified modules that were highly correlated/anti-correlated with the pathological model that were partially shared across transcriptome and translatome (RNA1, RNA3, and RNA4 overlap with Ribo1, Ribo2 and Ribo4; RNA14, RNA15, and RNA17 overlap with Ribo14, Ribo16, and Ribo17) (Figure 4A). These modules may represent ubiquitous processes and mechanisms of response to stress. Five RNA clusters were found to be highly preserved, and six moderately preserved, at the translatome level (Figure 4B). Six more RNA modules, one of which was activated (RNA5) after pressure overload in the pathological model, two of which were repressed (RNA13 and RNA16), as well as RNA7, RNA8, and RNA11, which showed differential activation/repression at 2d and 2w, had no or little gene overlap with translatome modules, and did not have a preserved network structure, suggesting that the transcriptome does not capture all key changes occurring in the heart during early hypertrophy.

To uncover how the heart transcriptome and translatome networks are rewired in response to stress, we highlighted genes which had the most dynamic neighborhoods. The Dn score, or dynamic neighborhoods score, captures changes in connectivity of a gene, even when its intramodular connectivity remains similar in RNA-seq and Ribo-seq networks, and is thus better suited than DC to highlight hub genes associated with potential regulatory mechanisms. The most rewired genes all belonged to the top preserved modules (Figure 4C). Among these, we highlighted two candidates, *Fam210a* and *Psmd12*, identified earlier (Supplementary Figure 6), which were strongly rewired, and whose immediate interactors were enriched in different GO terms between RNA-seq and Ribo-seq networks (Figure 4D and Supplementary Figure 9). *Fam210a* is a conserved transmembrane protein localized in the mitochondria, containing a mitochondrial targeting signal peptide (MitoCarta2.0 mouse), a DUF1279 (Domain of Unknown Function) domain with a transmembrane peptide, and a coiled coil at the C-terminus (InterPro). It is mostly expressed in the heart [1.72534] and skeletal muscle [1.57137] (Standardized values, BioGPS Mouse Cell Type and Tissue Gene Expression Profiles), and is thought to play a role in modulating muscle and bone biology (Tanaka et al., 2018), but its function in the heart and its molecular mechanisms are unknown. *Psmd12*, encoding the non-ATPase regulatory subunit 12 of the 26S proteasome, is better characterized. The 26S proteasome is a multiprotein complex involved in the ATP-dependent degradation of ubiquitinated proteins, and thus plays a key role in protein homeostasis. *Psmd12* is associated with several pathways, including Regulation of Apoptosis, Stabilization of p53, and p53-Dependent/Independent G1 DNA Damage Response (WikiPathways, Reactome). The tumor suppressor Trp53 (p53) regulates cell growth and fate, and its role in the heart is well-known. Psmd12 is also associated with inflammation [2.13579] and hypertrophy [2.10896] (Standardized values, CTD Comparative Toxicogenomics Database). Overall, module-eigengene association to disease phenotypes has led to the identification of highly rewired hub genes that may function as drivers of cardiac remodeling. These hub genes are potentially involved in related, but different molecular pathways or functions, suggesting some form of translational control that may not be immediately apparent from DTE analyses.

## 4. Discussion

In this study, a model of left ventricular pressure overload was used to mimic hypertrophy induced by systemic hypertension and aortic stenosis, and compared with a physiological model of exercise-induced cardiac growth. Transcriptional and translational co-expression networks uncovered *in vivo* changes in the heart occurring within 2 weeks of a transverse aortic constriction (TAC) surgery, revealing the complexity of the organization as well as unappreciated genes that may act as key drivers of the hypertrophic response.

Physiologic and pathophysiologic stimuli act upon the cell membrane and work their way through various cascades to mediate gene expression, translational control and protein levels (Haque and Wang, 2017). As expected, pressure overload was associated with profound changes in the composition of the extracellular matrix (ECM), which were reflected by a sub-clustering and a synchronized expression dynamics of ECM-, and cytoskeletal-related genes, in both transcriptome and translatome networks (Figures 2, 3, Supplementary Figures 4, 5, and Supplementary Table 4). A marked upregulation of genes encoding ECM proteins has previously been observed during the transition from stable cardiac hypertrophy to heart failure (Boluyt et al., 1994). Our results indicate that concerted dynamic changes occur early *in vivo* after stimuli, and are likely to be implicated in transducing molecular signals driving the maladaptive response. Concurrently to these observations, modules anti-correlated to the pathological model showed a diminished expression or altered contribution of mitochondrial, electron transport complex and oxidative phosphorylation genes. These modules (1–4, and 14, 15, 17) were also among the most preserved (Figure 4), suggesting the existence of stable sub-network structures, which could be associated with ubiquitous mechanisms of response to stress, although genes associated with these may or may not show significant changes in translational/transcriptional efficiency (Supplementary Figures 6, 7). Co-expression network and differential translational-efficiency (DTE) analyses are based on different assumptions (Langfelder and Horvath, 2008, 2012). In co-expression networks, the top genes are the most connected genes, based on the correlation structure.

In this study, we identified a number of hub genes that may function as molecular drivers of cardiac remodeling, many of which were recently described for their putative role in myofiber hypertrophy, cardiac inflammation or injury, such as *Gsk3a* (Ribo2) (Sugden et al., 2008; Zhou et al., 2016), *Cand2* (Ribo2) (Sandmann et al., 2018), *Rptor* (Ribo9) (Shende et al., 2011), *Lonp1* (Ribo9) (Venkatesh et al., 2019), *Ubr4* (Ribo9) (Hunt et al., 2019), *C5ar1* (RNA10) (Natarajan et al., 2018), *S100a4* (RNA10) (Doroudgar et al., 2016), or *Phb2* (Ribo13) (Wu D. et al., 2020). We also identified hub genes in the transcriptional network that were rewired in the translational network, and associated with semantically different subsets of enriched terms (Figure 4). Notably, we highlighted the presence of two hub genes that were rewired under hypertrophic stimuli, *Fam210a* (RNA15, Ribo16), and *Psmd12* (RNA17, Ribo16). *Fam210a*, a gene of previously unknown function, has been described as a musculoskeletal modulator (Tanaka et al., 2018). In humans, a prior study reported that *Fam210a* (*C18orf19*) was the strongest candidate partner protein of *Atad3a* (ATPase Family AAA Domain Containing 3A), which was also found in the same modules (RNA15 and/or Ribo16), along with 60 (out of 153) interacting proteins identified by Orbitrap MS analysis and quantified by SILAC labeling (He et al., 2012). *Atad3a* is essential for mitochondrial metabolism and translation, and has been implicated in several processes in mitochondria. More recent work, which we discovered while this manuscript was under review, has shown how the miR-574-*Fam210a* axis regulates mitonchondrial-encoded protein expression in cardiac pathological remodeling (Wu J. et al., 2020). Taken together, these results suggest that *Fam210a* could modulate translation of mitochondrial-encoded electron-transport chain proteins, and play a yet undescribed role in cardiac muscle adaptation and growth. The biological importance of *Psmd12* as a scaffolding subunit in proteasome function has been described earlier in the context of neuronal development, but remains un-documented in the heart. The ubiquitin proteasome system (UPS) is critical in preventing accumulation of damaged and misfolded proteins, and has been implicated in a number of cardiac proteinopathies and heart failure (Pagan et al., 2013; Cacciapuoti, 2014; Maejima, 2020). Our results support the existence of transcriptional/translational regulatory processes affecting the or affected by proteasome function in the pathogenesis of cardiac hypertrophy.

In summary, these results highlight the organization of distinct molecular processes into sub-networks of co-expressed genes, and describe how transcriptome and translatome signatures are orchestrated into functional modules associated with the early stages of cardiac remodeling. Our results constitute a valuable resource to study *in vivo* cardiac regulatory networks, and a first step toward the identification and characterization of novel proteins involved in cardiac remodeling, hypertrophy and heart failure.

## Data Availability Statement

The data generated for this study have been deposited in NCBI's Sequence Read Archive through the BioProject accession numbers PRJNA484227 and PRJNA543399. All raw counts and translation events used in this study are available as Supporting Information. RP-BP is publicly available at https://github.com/dieterich-lab/rp-bp under the MIT License. The code for generating co-expression networks is available as Supplementary Information.

## Ethics Statement

The animal study was reviewed and approved by Ethikkommission der Med. Fakultät Heidelberg.

## Author Contributions

EB and CD conceptualized the project. EB conducted formal analyses and interpretation, maintained the software and was in charge of the original draft preparation. CD was in charge of project administration, supervision, contributed to resources, data analysis and interpretation. SD and MV contributed to resources, data acquisition, and investigation. ER, LJ, and TH performed animal experiments. HK provided funding acquisitions. EB, CD, SD, and MV contributed to review and editing. All authors contributed to the article and approved the submitted version.

## Conflict of Interest

The authors declare that the research was conducted in the absence of any commercial or financial relationships that could be construed as a potential conflict of interest.

## Supplementary Material

Supplementary Figure 1 A survey of active translation in 15 mouse hearts representing models of cardiac remodeling.

Supplementary Figure 2 A survey of active translation in 15 mouse hearts: open reading frames (ORFs) translated in cardiac cells.

Supplementary Figure 3 Co-expression networks of transcriptional and translational regulation in cardiac remodeling: schematic workflow.

Supplementary Figure 4 Translatome (RNA) network modules were clustered to assess relatedness based on correlation of co-expressed eigengenes.

Supplementary Figure 5 Translatome (Ribo) network modules were clustered to assess relatedness based on correlation of co-expressed eigengenes.

Supplementary Figure 6 Hub genes provide a different molecular signature for differentiating physiological and pathological cardiac remodeling.

Supplementary Figure 7 Hub genes provide a different molecular signature for differentiating physiological and pathological cardiac remodeling.

Supplementary Figure 8 Differential connectivity and differential translational-efficiency.

Supplementary Figure 9 Rewired genes are associated with different biological processes, molecular functions and cellular components in transcriptome vs. translatome.

Supplementary Table 1 Open reading frame predictions per sample, all categories, Rp-Bp output.

Supplementary Table 2 Open reading frame predictions, unique (translated genes used in this study).

Supplementary Table 3 Transcriptome and translatome read counts used as input for network analyses.

Supplementary Table 4 Transcriptome and translatome co-expression modules.

Supplementary Table 5 Gene ontology enrichment of transcriptome and translatome co-expression modules.

Supplementary Table 6 Transcriptome and translatome co-expression modules correlation association and significance.

Supplementary Table 7 Extended differential translational-efficiency (DTE) results.

Supplementary Information Code for generating co-expression networks.
