# Corrigendum: A Multi-Network Comparative Analysis of Transcriptome and Translatome Identifies Novel Hub Genes in Cardiac Remodeling

**DOI:** 10.3389/fgene.2021.706542

**Published:** 2021-06-17

**Authors:** Etienne Boileau, Shirin Doroudgar, Eva Riechert, Lonny Jürgensen, Thanh Cao Ho, Hugo A. Katus, Mirko Völkers, Christoph Dieterich

**Affiliations:** ^1^Section of Bioinformatics and Systems Cardiology, Klaus Tschira Institute for Integrative Computational Cardiology, Heidelberg, Germany; ^2^Department of Internal Medicine III (Cardiology, Angiology, and Pneumology), University Hospital Heidelberg, Heidelberg, Germany; ^3^DZHK (German Centre for Cardiovascular Research), Partner Site Heidelberg/Mannheim, Berlin, Germany

**Keywords:** cardiovascular, cardiac hypertrophy, transcription/RNA-seq, translation/Ribo-seq, co-expression networks

There is an error in the Data Availability Statement. The correct number for **NCBI's Sequence Read Archive BioProject** associated with this work is **PRJNA543399**. The Data Availability Statement should read:

The data generated for this study have been deposited in NCBI's Sequence Read Archive through the BioProject accession numbers PRJNA484227 and PRJNA543399. All raw counts and translation events used in this study are available as Supporting Information. RP-BP is publicly available at https://github.com/dieterich-lab/rp-bp under the MIT License. The code for generating co-expression networks is available as Supplementary Information.

The authors apologize for this error and state that this does not change the scientific conclusions of the article in any way. The original article has been updated.

