# Supplementary material for: A Multi-Network Comparative Analysis of Transcriptome and Translatome Identifies Novel Hub Genes in Cardiac Remodeling
Source: Front Genet. 2020 Nov 16;11:583124. doi: 10.3389/fgene.2020.583124 (PMC7701244; doi:10.3389/fgene.2020.583124)
Supplement: Supplementary Figure 1 — A survey of active translation in 15 mouse hearts representing models of cardiac remodeling. [file Data_Sheet_1.PDF]

Supplementary Material

1 SUPPLEMENTARY TABLES AND FIGURES

1.1 Figures

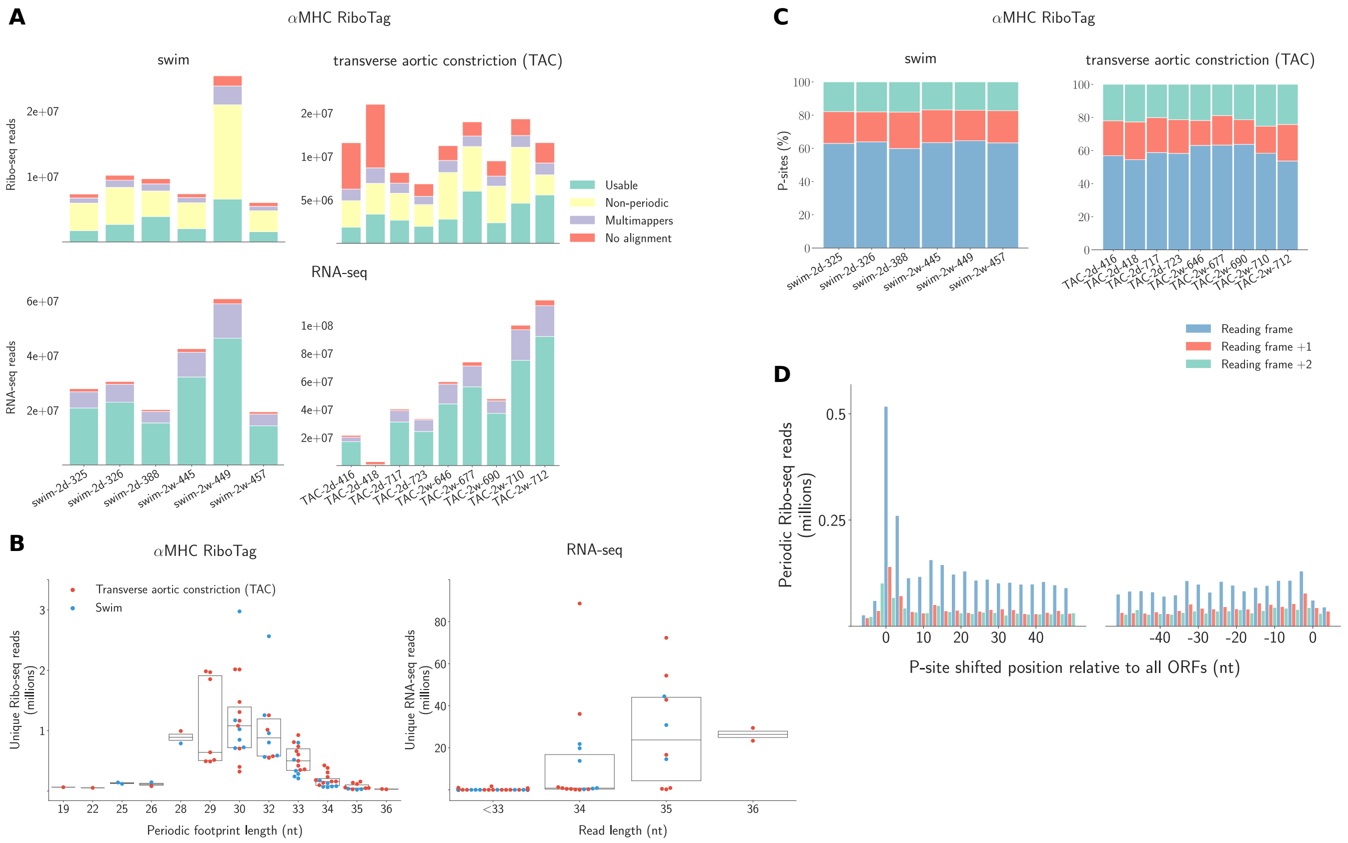

Figure S1. A survey of active translation in 15 mouse hearts representing models of cardiac remodeling. (A) Bar plots showing the number of raw sequenced reads without alignment, mapping to more than one genomic position and, for Ribo-seq, without evidence of periodicity, as determined by RP-BP, for all 15 mouse hearts, per experimental model and protocol. Usable reads are unique Ribo-seq reads with evidence of periodicity, or uniquely mapped RNA-seq reads. The raw sequenced reads of poor quality, mapped to tRNAs or ribosomal RNA (rRNA) are removed and not shown. (B) Swarmplot showing the number of reads of a given periodic footprint length, for Ribo-seq, as determined by RP-BP, for all 15 mouse hearts, per experimental model and protocol. RNA-seq reads were trimmed from the 3' end after adapter removal, such that the read length before alignment did match the maximum periodic fragment length of the corresponding Ribo-seq sample. (C) Evidence of ribosome periodicity as the percentage of footprints that are in-frame (blue) with respect to all identified ORFs, for all 15 mouse hearts, per experimental model and protocol. (D) Bar plot displaying the aggregated codon periodicity for all 15 mouse hearts across all ORFs.

A

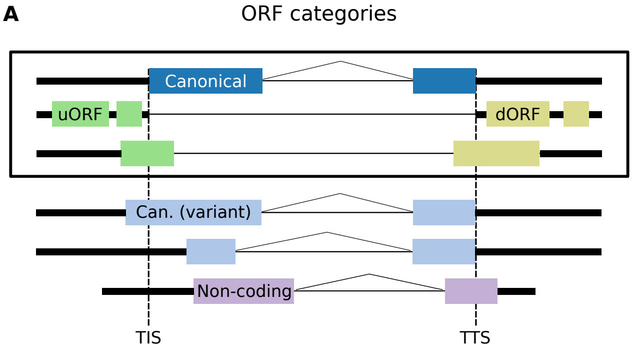

B

Category	Median size (aa)	Translated	
		>100 aa	sORF
Canonical	486	16210	429
Upstream ORF	22	93	2604
Downstream ORF	57	211	725
Can. (variant)	343	2352	666
Non-coding	95	842	882

Figure S2. A survey of active translation in 15 mouse hearts: open reading frames (ORFs) translated in cardiac cells. To generate a comprehensive catalog of translation events in the heart, we performed *in vivo* Ribo-seq using the RiboTag mouse (Fig. S1), combining the resolution of Ribo-seq with the cell-specificity of RiboTag. To search for actively-translated ORFs, we used RP-BP, based on Ensembl release 96, as described in the manuscript. (A) The classification of reading frames at the predicted translation initiation site was done relative to the annotated coding transcripts. Canonical variants include 'N-terminus' extended and truncated sequences as well as small out-of-frame coding sequences. Upstream (uORF) and downstream (dORF) open reading frames can overlap the primary coding sequence. 'Non-coding ORFs' originate from transcripts not annotated as coding (any non-coding, processed transcript or pseudogene). For all analyses, including the construction of gene co-expression networks, we only used evidence of translation from canonical, upstream and/or downstream ORFs (boxed ORFs above). (B) Table indicating the median size, and the number of translated ORFs, for ORFs > 100 aa and small ORFs (< 100 aa). The resulting set of translated ORFs comprises 25,014 unique sequences, that were found with features of active translation in at least one of the conditions, according to the probabilistic graphical models (Supplementary Table S1). For all analyses, inclusing the construction of gene co-expression networks, predicted ORFs were further required to have evidence of at least 10 in-frame P-sites, in at least 3 samples, and their host gene was also required to be annotated in APPRIS. The final set of ORFs thus comprises 11,728 unique ORFs (canonical, uORF, and dORF), see Supplementary Table S2. TIS=annotated translation initiation site, TTS=annotated translation termination site, sORF=small ORF.

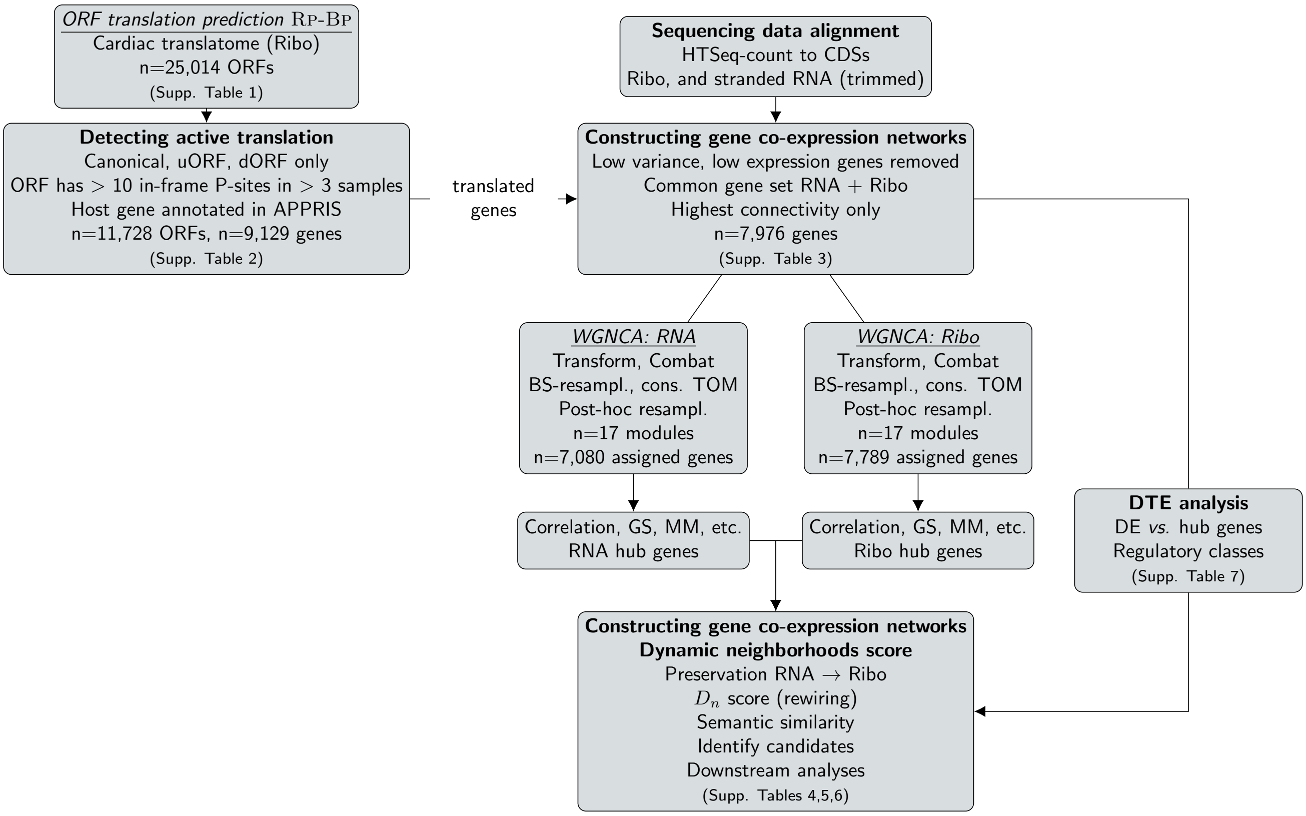

Figure S3. Co-expression networks of transcriptional and translational regulation in cardiac remodeling: schematic workflow. Using the set of translated genes (cardiac translatome), we constructed gene co-expression networks independently for RNA-seq and Ribo-seq counts. Differential translational-efficiency (DTE) analyses were performed, and differentially regulated (DE) genes were compared to hub genes, obtained from co-expression analyses. Based on preservation statistics, rewiring metrics, and semantic similarities, top candidate genes were identified. The bold text refer to the section of the same name in the manuscript (MATERIALS AND METHODS), where detailed information is presented. Data generated and provided as supplementary material is indicated in relevant boxes. ORF=open reading frame, uORF=upstream ORF, dORF=downstream ORF, CDS=coding sequence, BS-resampl.=bootstrap resampling, cons. TOM=consensus topological overlap (TO) matrix, GS=gene significance, MM=module membership.

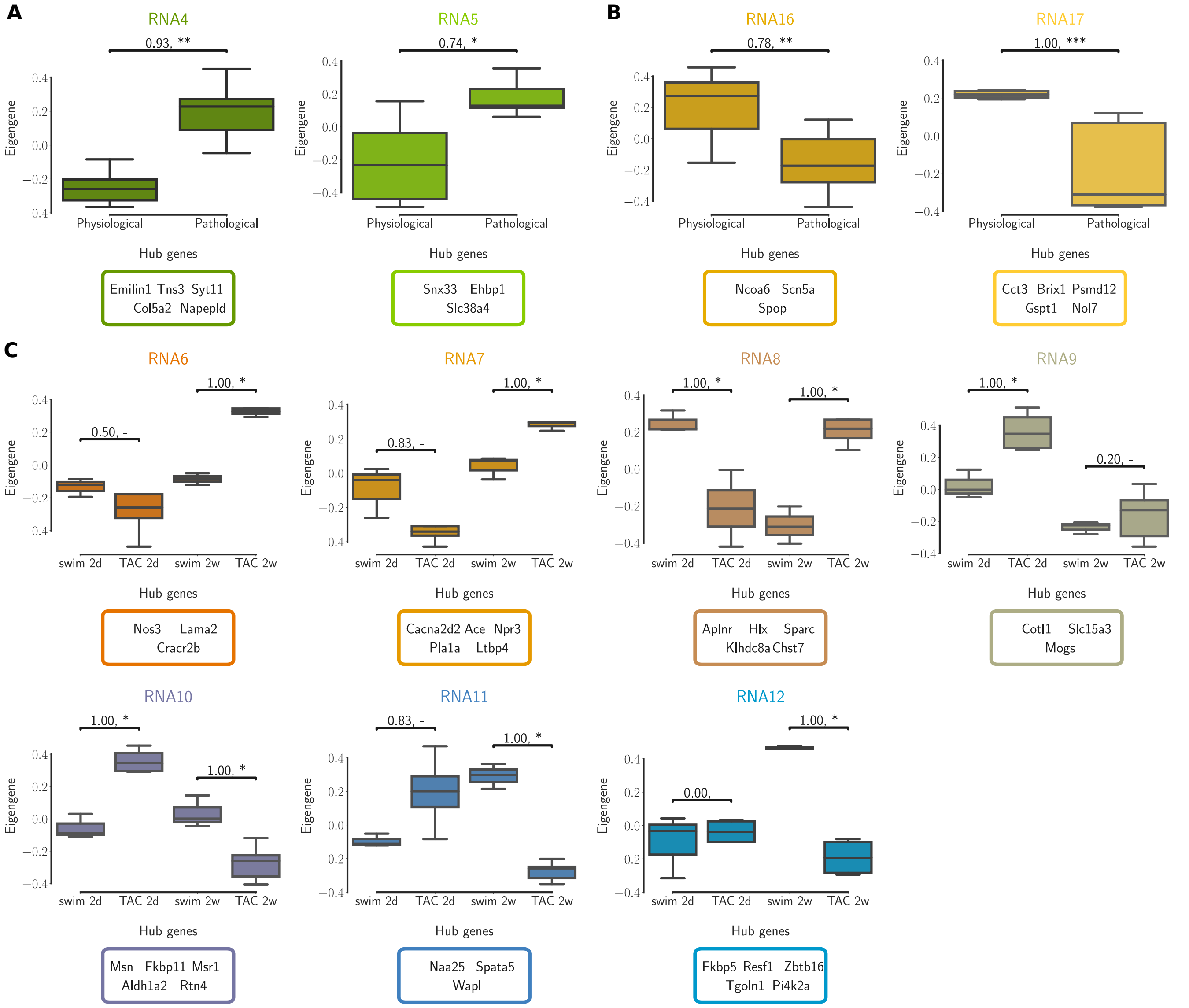

Figure S4. Transcriptome (RNA) network modules were clustered to assess relatedness based on correlation of co-expressed eigengenes. (A) Selected cluster expression profiles and top ranked hub genes that are positively correlated with pathological cardiac remodeling. (B) Selected cluster expression profiles and top ranked hub genes that are negatively correlated with pathological cardiac remodeling (*i.e.* positively correlated with the physiological model). (C) Cluster expression profiles and top ranked hub genes that are differentially correlated with pathological cardiac remodeling at different time points. Boxplots are displayed for all time points in the physiological and pathological groups (A, B), and for each separate time points in the swim and TAC groups (C). Significance was measured using a one-sided Mann-Witney U-test, at a threshold of 0.05 (** = <0.01, ***= <0.001, p-values may be affected by the small sample size, for this reason we also report effect size using non-directional rank-biserial correlation). Up to five hub genes are shown.

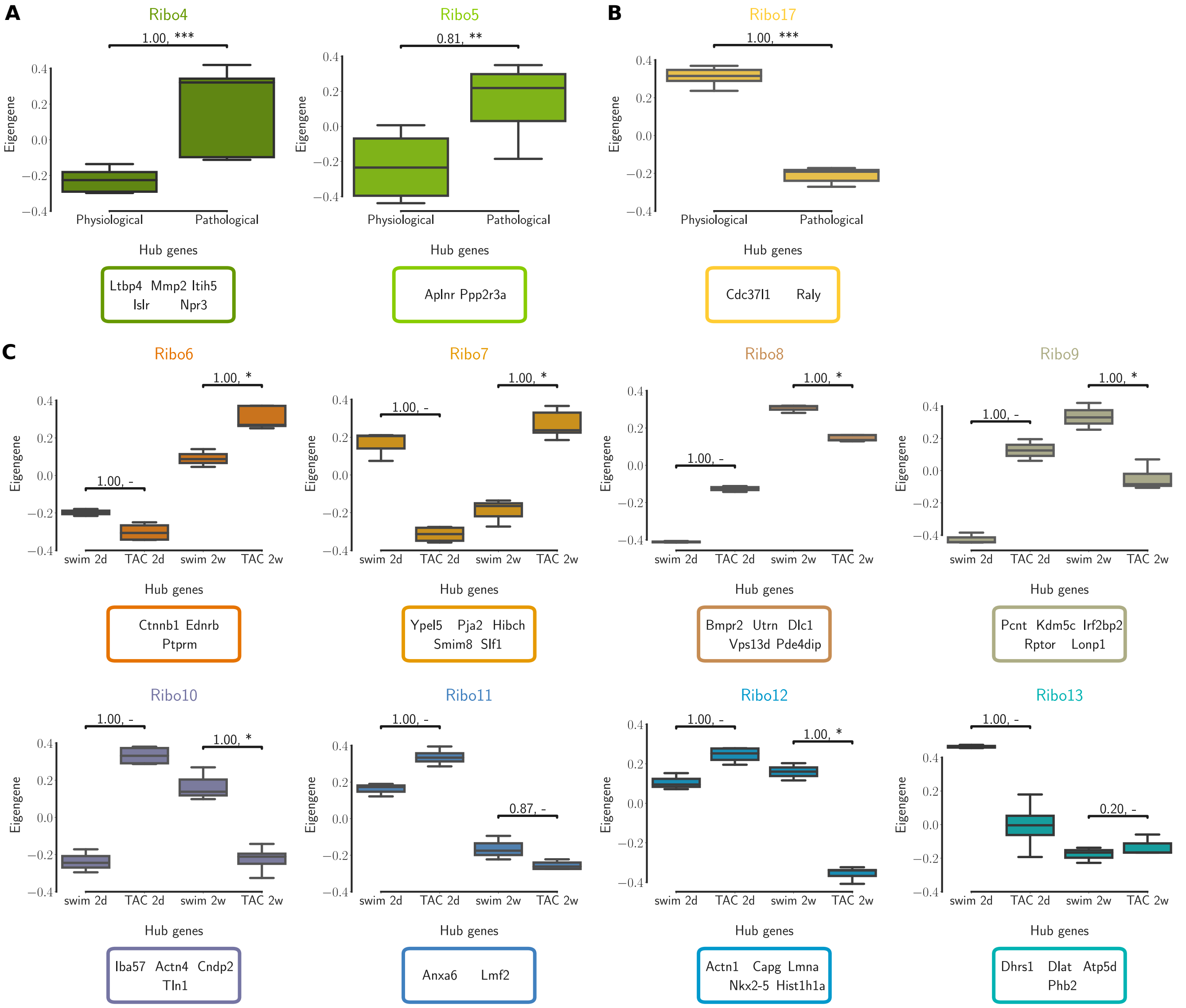

Figure S5. Translatome (Ribo) network modules were clustered to assess relatedness based on correlation of co-expressed eigengenes. (A) Selected cluster expression profiles and top ranked hub genes that are positively correlated with pathological cardiac remodeling. (B) Selected cluster expression profiles and top ranked hub genes that are negatively correlated with pathological cardiac remodeling (*i.e.* positively correlated with the physiological model). (C) Cluster expression profiles and top ranked hub genes that are differentially correlated with pathological cardiac remodeling at different time points. Boxplots are displayed for all time points in the physiological and pathological groups (A, B), and for each separate time points in the swim and TAC groups (C). Significance was measured using a one-sided Mann-Witney U-test, at a threshold of 0.05 (** = <0.01, ***= <0.001, p-values may be affected by the small sample size, for this reason we also report effect size using non-directional rank-biserial correlation). Up to five hub genes are shown.

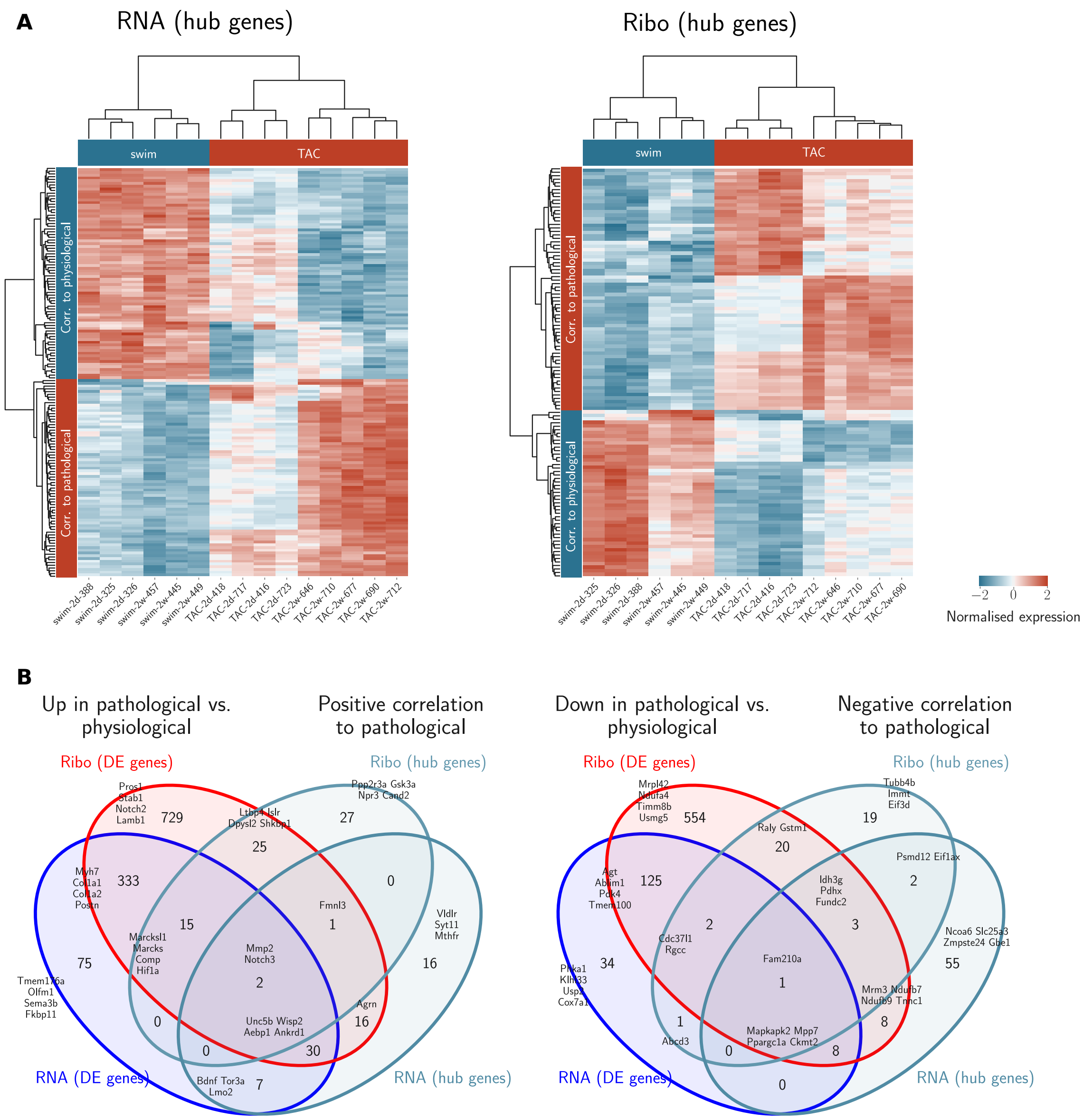

Figure S6. Hub genes provide a different molecular signature for differentiating physiological and pathological cardiac remodeling. (A) Expression profile of all hub genes associated with co-expression clusters having a positive or a negative correlation to pathological phenotypes after unsupervised hierarchical clustering, showing a separation between samples of the physiological and pathological models, and between positively and negatively correlated eigengene-associated cluster. Hubs genes are from modules RNA1 to RNA5, RNA13 to RNA17, and from Ribo1 to Ribo5, Ribo14 to Ribo17. (B) *Left* Venn diagram showing the overlap between differentially up-regulated genes at the transcriptional and translational level in the pathological model, and hub genes in the RNA-seq and Ribo-seq co-expression modules with positive correlation to pathological phenotypes. *Right* Venn diagram showing the overlap between differentially down-regulated genes at the transcriptional and translational level in the pathological model, and hub genes in the RNA-seq and Ribo-seq co-expression modules with negative correlation to pathological phenotypes. Transcriptionally-regulated genes are DTE in RNA, or RNA+Ribo; translationally-regulated genes are DTE in Ribo, RNA+Ribo, or TE (FDR < 0.05, fold change $> \log_2(1.2)$). Top four differentially regulated genes are indicated. For all subsets intersecting hub genes, up to four selected hub genes among the top ranked genes ($<$15) are also indicated. DE=Differentially expressed.

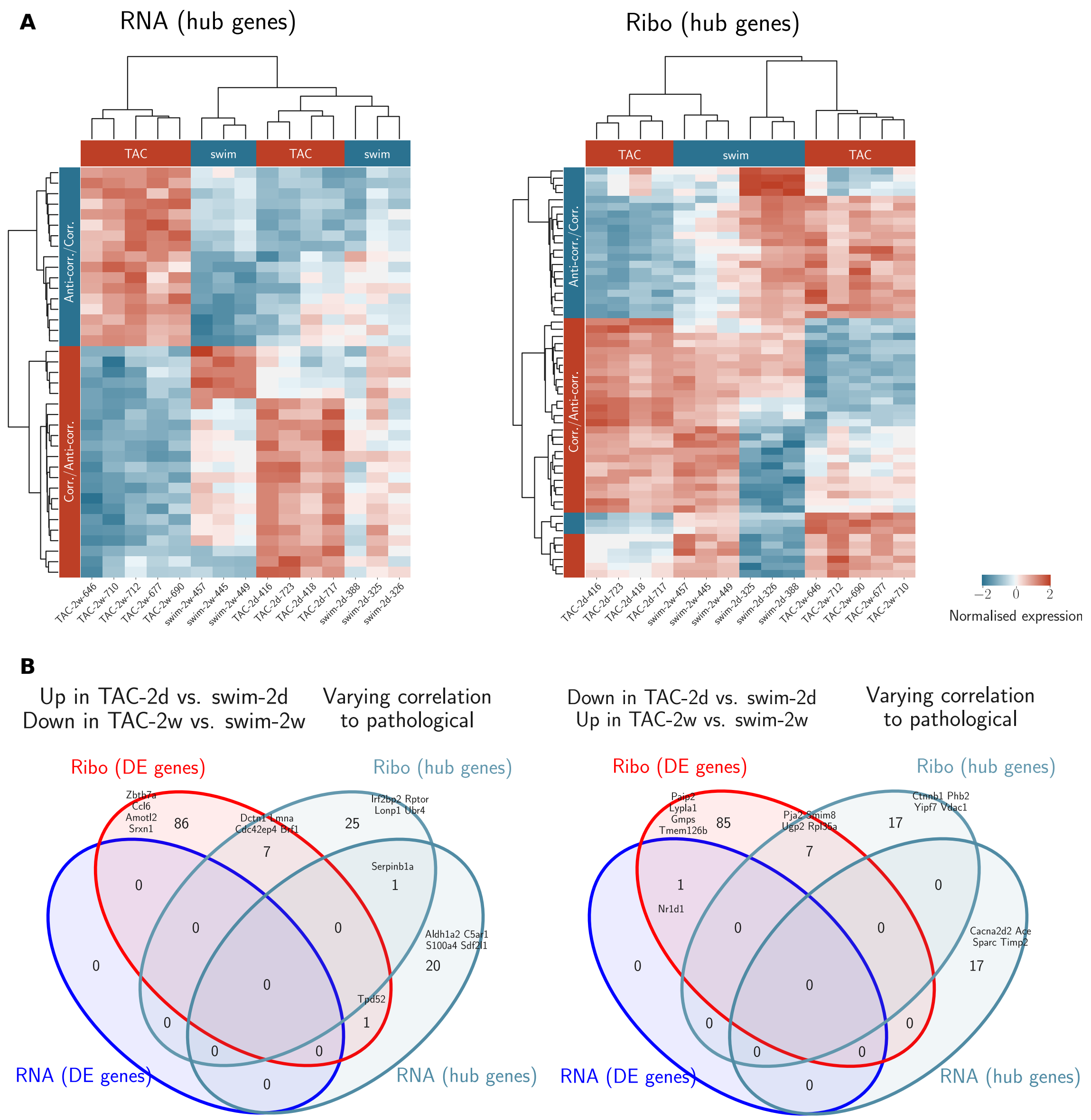

Figure S7. Hub genes provide a different molecular signature for differentiating physiological and pathological cardiac remodeling. (A) Expression profile of all hub genes associated with co-expression modules having a varying correlation between 2d and 2w to pathological phenotypes after unsupervised hierarchical clustering, showing a separation between samples of the physiological and pathological models at 2d and 2w, and between the eigengene correlation (Corr./Anti-corr.=positively correlated at 2d and negatively correlated at 2w to pathological phenotypes, Anti-corr./Corr.=negatively correlated at 2d and positively correlated at 2w to pathological phenotypes). Hubs genes are from modules RNA6 to RNA12, and from Ribo6 to Ribo13. (B) *Left* Venn diagram showing the overlap between differentially up-regulated genes at 2d and down-regulated genes at 2w, at the transcriptional and translational level, in the pathological model, and hub genes in the RNA-seq and Ribo-seq co-expression modules with positive correlation at 2d and negative correlation at 2w to pathological phenotypes (Corr./Anti-corr.). *Right* Venn diagram showing the overlap between differentially down-regulated genes at 2d and up-regulated genes at 2w, at the transcriptional and translational level, in the pathological model, and hub genes in the RNA-seq and Ribo-seq co-expression modules with negative correlation at 2d and positive correlation at 2w to pathological phenotypes (Anti-corr./Corr.). Top differentially regulated genes are indicated. Transcriptionally-regulated genes are DTE in RNA, or RNA+Ribo; translationally-regulated genes are DTE in Ribo, RNA+Ribo, or TE (FDR < 0.05, fold change $> \log_2(1.2)$). Top four differentially regulated genes are indicated. For all subsets intersecting hub genes, up to four selected hub genes among the top ranked genes (<15) are also indicated. DE=Differentially expressed.

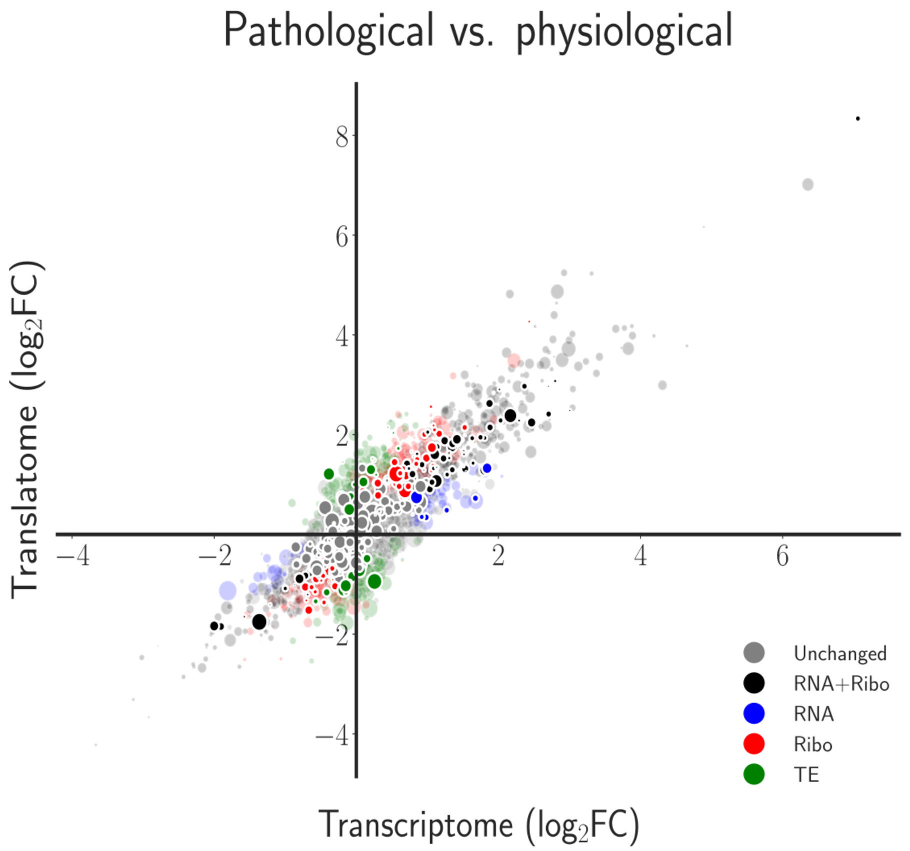

Figure S8. Differential connectivity and differential translational-efficiency. Scatter plot showing the log-fold changes in the RNA-seq and Ribo-seq for the differential translational-efficiency (DTE) analysis. Dot size reflects the absolute value of the ratio of intramodular connectivities between RNA-seq and Ribo-seq, also referred to as differential connectivity (DC). Dark-colored dots are the RNA and Ribo hub genes across all clusters. A large number of unchanged genes in DTE show a higher differential connectivity. See also Supplementary Table S7.

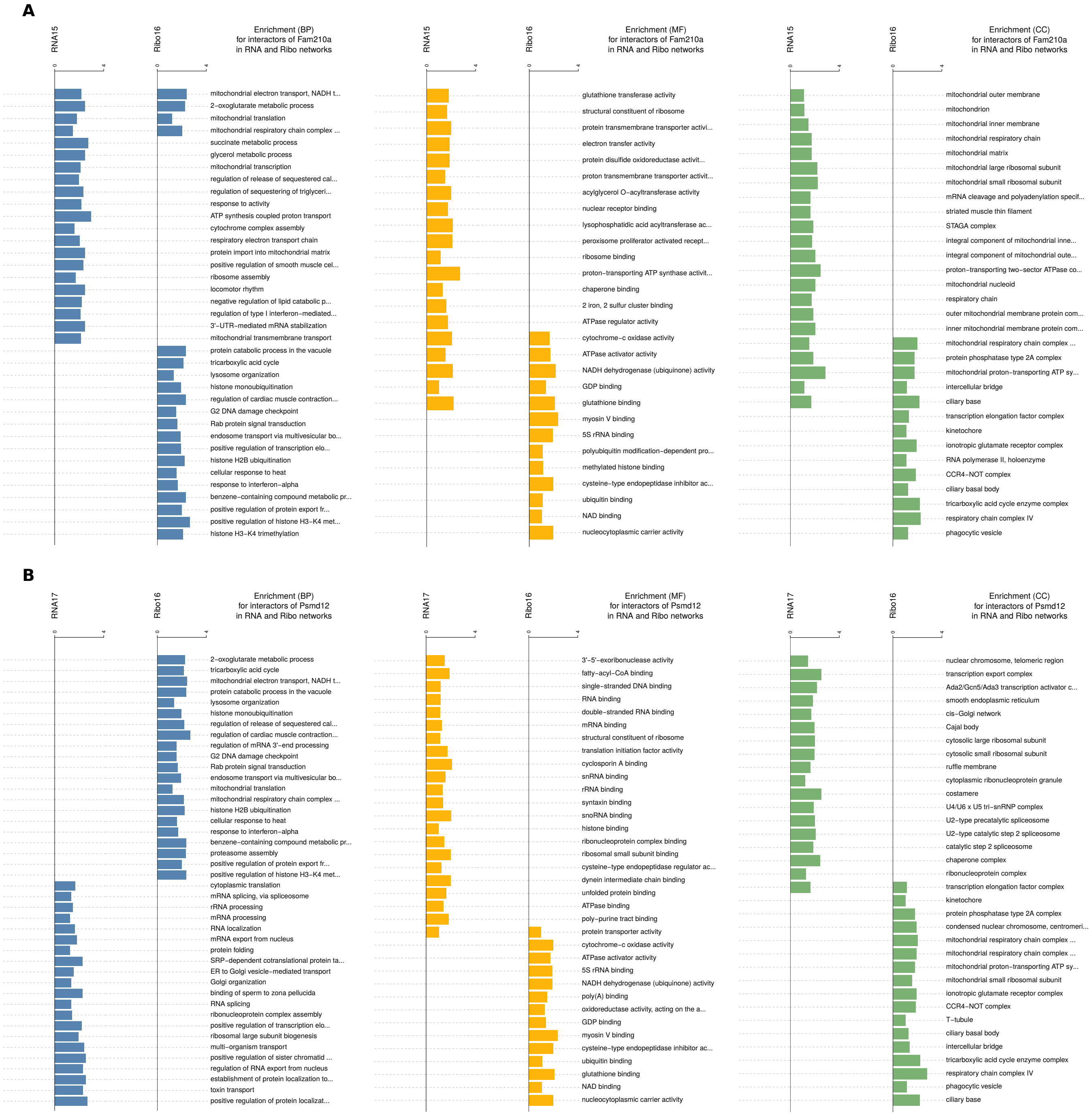

Figure S9. Rewired genes are associated with different biological processes, molecular functions and cellular components in transcriptome *vs.* translatome. Gene enrichment (GO) analysis based on immediate interactors of (A) Fam210a, and (B) Psmd12. The top significant terms (p-value < 0.05) are shown with $\log_2$ enrichment values (>1) on the x-axis. The set of all translated genes was used as background.
